# Supplementary material for: Short-Term Head-Out Whole-Body Cold-Water Immersion Facilitates Positive Affect and Increases Interaction between Large-Scale Brain Networks
Source: Biology (Basel). 2023 Jan 29;12(2):211. doi: 10.3390/biology12020211 (PMC9953392; doi:10.3390/biology12020211)
Supplement: Supplementary file 1 [file biology-12-00211-s001.zip › biology-2138490-supplementary.pdf]

biology

MDPI

Supplementary Materials

Short-term head-out whole-body cold-water immersion facilitates positive affect and increases interaction between large-scale brain networks

Ala Yankouskaya [1,*], Ruth Williamson [2], Cameron Stacey [1], John James Totman [3] and Heather Massey [4]

```
DefaultMode.MPFC (1,55,-3)
DefaultMode.LP (L) (-39,-77,33)
DefaultMode.LP (R) (47,-67,29)
DefaultMode.PCC (1,-61,38)
SensoriMotor.Lateral (L) (-55,-12,29)
SensoriMotor.Lateral (R) (56,-10,29)
SensoriMotor.Superior  (0,-31,67)
Visual.Medial (2,-79,12)
Visual.Occipital (0,-93,-4)
Visual.Lateral (L) (-37,-79,10)
Visual.Lateral (R) (38,-72,13)
Salience.ACC (0,22,35)
Salience.AInsula (L) (-44,13,1)
Salience.AInsula (R) (47,14,0)
Salience.RPFC (L) (-32,45,27)
Salience.RPFC (R) (32,46,27)
Salience.SMG (L) (-60,-39,31)
Salience.SMG (R) (62,-35,32)
DorsalAttention.FEF (L)  (-27,-9,64)
DorsalAttention.FEF (R)  (30,-6,64)
DorsalAttention.IPS (L)  (-39,-43,52)
DorsalAttention.IPS (R)  (39,-42,54)
FrontoParietal.LPFC (L)  (-43,33,28)
FrontoParietal.PPC (L)  (-46,-58,49)
FrontoParietal.LPFC (R)  (41,38,30)
FrontoParietal.PPC (R)  (52,-52,45)
Language.IFG (L) (-51,26,2)
Language.IFG (R) (54,28,1)
Language.pSTG (L) (-57,-47,15)
Language.pSTG (R) (59,-42,13)
Cerebellar.Anterior (0,-63,-30)
Cerebellar.Posterior (0,-79,-32)
```

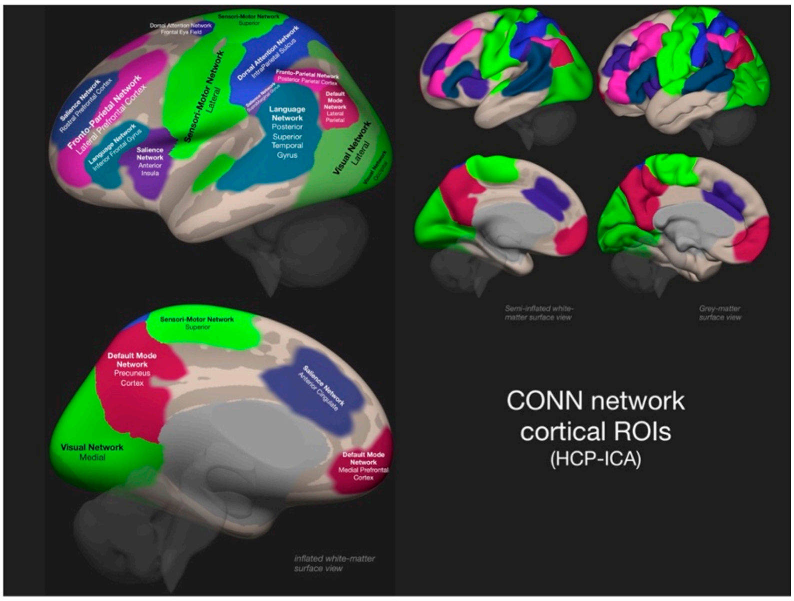

Figure S1. Large scale brain networks derived from CONN's atlas (Whitfield-Gabrieli & Nieto-Castanon, 2012) [42].

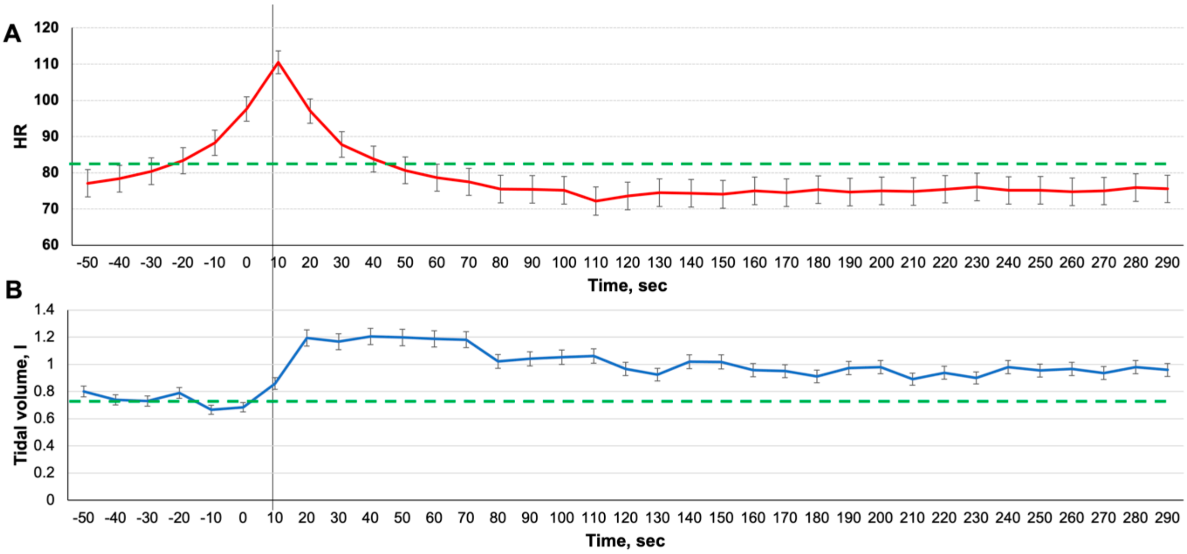

Figure S2. Means heart rate (A) and tidal volume (the amount of air that moves in or out of the lungs with each respiratory cycle, B) 60 sec prior CWI (PRE-CWI), CWI (start of cold-water

immersion). The X-axes represents time of CWI. Error bars represent SEM at each time point. Dashed green lines showed the means of the baseline measurements.

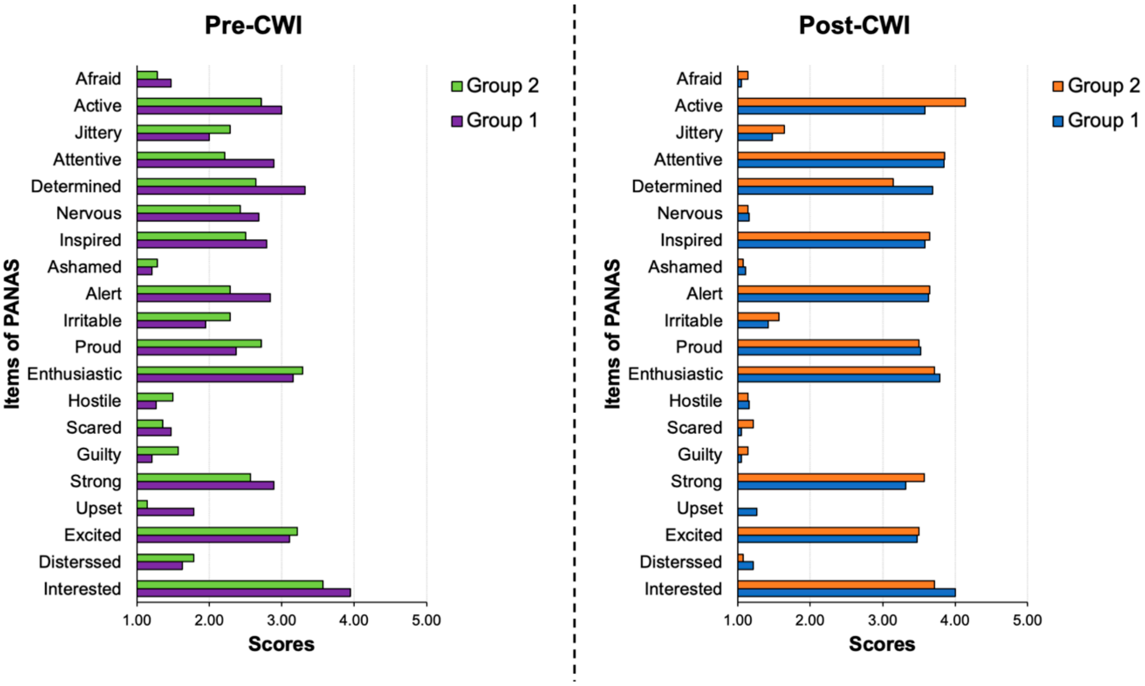

Figure S3. Clustered bar plots of individual items of PANAS for Pre-Post-CWI for Group 1 (participants experiences with MRI/fMRI and Group 2 (participants naïve to MRI/fMRI).

Table S1. Individual PANAS scores for Pre-CWI and Post-CWI.

ID	Pre-positive	Post-positive	Pre-negative	Post-negative
1	15	40	29	10
2	24	28	19	13
3	32	39	23	22
4	28	31	23	20
5	31	31	14	11
6	21	37	18	10
7	32	47	17	13
8	38	39	17	10
9	16	33	15	13
10	38	42	18	12
11	28	46	12	10
12	32	39	24	12
13	33	37	12	12
14	18	34	14	10
15	26	33	24	12
16	26	33	11	10
17	32	41	13	11
18	32	32	13	10
19	34	41	15	16
20	39	38	15	14
21	22	24	12	10
22	21	34	25	14
23	28	36	10	10

24	21	27	17	10
25	34	45	11	10
26	30	31	17	10
27	**43**	**39**	18	12
28	38	39	13	10
29	39	40	18	11
30	**22**	**20**	18	12
31	27	46	**11**	**11**
32	39	41	20	15

Table S2. Results of Wilcoxon's signed rank test on individual items of PANAS. W is defined as the smaller of W+ (sum of the positive ranks) and W- (sum of the negative ranks). BF_{10} - the Bayes factor in favour of H1 over H0.

**Measures [Pre – Post]**	**W**	**z**	***p***	**BF_{10}**
Interested	43.00	-0.59	0.55	0.26
Active	18.00	-3.25	<.001	79.51
Afraid	48.00	2.09	0.04	1.73
Alert	32.00	-3.21	.001	83.24
Ashamed	10.00	1.83	.09	0.44
Attentive	8.00	-4.06	<.001	1155.12
Determined	31.00	-1.91	.05	1.44
Distressed	99.00	2.92	.002	22.84
Enthusiastic	28.00	-2.50	.009	5.42
Exited	37.00	-1.85	.05	0.99
Guilty	28.00	2.37	.02	1.35
Hostile	34.50	1.42	.16	0.35
Inspired	6.00	-3.69	<.001	445.06
Irritable	84.00	2.69	.007	4.55
Jittery	117.00	2.56	.009	9.95
Nervous	406.00	4.62	<.001	26282.93
Proud	5.00	-3.51	<.001	1151.66
Scared	41.00	2.19	.03	1.19
Strong	64.50	-2.44	.01	7.08
Upset	45.00	2.67	.006	3.87

Table S3. The results of Network Based Statistic analysis with varied 'height' thresholds for contrast **[Post-CWI>Pre-CWI]** (p-value for each component was FWE corrected). NS denotes that a component did not survive the FWE correction.

**Height threshold (p<)**	**Connections comprising a component**	**Component statistics**		
		**mass**	**size**	**p-FWE value**
.05 - .02	NS			
.01 - .006	DMN.MPFC-SN.ACC<br>DMN.MPFC-SN.RPFC(L)<br>FP.PCC(R)-Vis.Lat(R)<br>FP.PCC(R)-DAN.IPS(R)<br>DMN.PCC -Vis.Lat (R)<br>DMN.MPFC-DMN.LP (L)<br>DMN.PCC-LP (R)	357	1	.003

	DMN.MPFC-SN.Ains (L)			
.005 - .002	DMN.MPFC-SN.ACC<br>DMN.MPFC-SN.RPFC(L)<br>FP.PCC(R)-Vis.Lat(R)<br>FP.PCC(R)-DAN.IPS(R)<br>DMN.PCC -Vis.Lat (R)<br>DMN.MPFC-DMN.LP (L)<br>DMN.PCC-LP (R)<br>DMN.MPFC-SN.Ains (L)	168.06<br><br>165.30	2	.013 - .001
.001 -.0004	DMN.MPFC-SN.ACC<br>DMN.MPFC-SN.RPFC(L)<br>FP.PCC(R)-Vis.Lat(R)<br>FP.PCC(R)-DAN.IPS(R)<br>DMN.MPFC-DMN.LP (L)<br>DMN.MPFC-SN.Ains (L)	165.30<br><br>126.78	2	< .001 - .001
.0005 - .00001	DMN.MPFC-SN.ACC<br>DMN.MPFC-SN.RPFC(L)<br>FP.PCC(R)-Vis.Lat(R)<br>FP.PCC(R)-DAN.IPS(R)<br>DMN.MPFC-DMN.LP (L)	126.78<br><br>151.22	2	< .001
.0000009	FP.PCC(R)-Vis.Lat(R)<br>FP.PCC(R)-DAN.IPS(R)	64.23	1	.03
.0000005	NS			

Table S4. The results of Network Based Statistic analysis with varied 'height' thresholds for contrast **[Post-CWI>Pre-CWI]*Changes in Positive Affect** (p-value for each component was FWE corrected). NS denotes that a component did not survive the FWE correction.

Height threshold (p<)	Connections comprising a component	Component statistics		
		mass	size	p-FWE value
.05 - .004	NS			
.003 - .00002	DMN.MPFC-SN.ACC<br>DMN.MPFC-SN.RPFC(L)	102.86	2	.019 - .00002
	FP.PCC(R)-Vis.Lat(R)<br>FP.PCC(R)-DAN.IPS(R)	96.62	2	0.03-0.0004
.00001 - .00007	DMN.MPFC-SN.ACC	86.11	1	.006 -.004
	FP.PCC(R)-Vis.Lat(R)<br>FP.PCC(R)-DAN.IPS(R)	96.62	2	
.00006 - .000002	FP.PCC(R)-DAN.IPS(R)	46.90	1	.004 - .04
.000001	NS			

Table S5. The results of Network Based Statistic analysis with varied 'height' thresholds for contrast **[Post-CWI>Pre-CWI]*Changes in Negative Affect** (p-value for each component was FWE corrected). NS denotes that a component did not survive the FWE correction.

Height threshold (p<)	Connections comprising a component	Component statistics		
		mass	size	p-FWE value
.05 - .03	Vis. Lat (R)<br>Vis. Lat (L)<br>Vis. Med	594.50	68	.02

	DAN.IPS (R)<br>DAN.IPS (L)<br>DAN.FEF (L)<br>SM.Superior<br>SN.RPFC (R)<br>SN.ACC<br>SN.AIns (R)<br>SN.AIns (L)<br>SN.SMG (L)<br>Language.IFG (R)<br>Language.pSTS (R)<br>Language.pSTS (L)<br>Language.IFG (L)<br>FP.LPFC (R)<br>FP.PPC (L)<br>FP.LPFC (L)<br>DMN.MPFC<br>DMN.PCC<br>DMN.LP (R)<br>DMN.LP (L)			
.02	FP.LPFC (R)<br>DMN.MPFC<br>DMN.PCC<br>DMN.LP (R)<br>DMN.LP (L)<br>DAN.IPS (R)<br>DAN.IPS (L)<br>DAN.FEF (L)<br>SM.Superior<br>Language.IFG (R)<br>SN.SMG (L)<br>SN.Ains (L)<br>SN.AIns (R)	387.72	30	.016
.01-.001	DMN.MPFC<br>DMN.PCC	154.50	2	.003
.0009-.0001	FP.PCC(R)->DAN.IPS(R)	46.90	1	.004 - .04
.000001	NS			
